# Editorial: The Role of Neurovascular Unit in Neurodegeneration

**DOI:** 10.3389/fncel.2022.870631

**Published:** 2022-04-19

**Authors:** Zhi Dong Zhou, Dennis Qing Wang, Eng-King Tan

**Affiliations:** ^1^Department of Research, National Neuroscience Institute, Singapore, Singapore; ^2^Neuroscience and Behavioural Disorders Programme (NBD), Duke-National University of Singapore Medical School, Singapore, Singapore; ^3^Zhujiang Hospital of Southern Medical University, Guangzhou, China; ^4^Department of Neurology, Singapore General Hospital, Singapore, Singapore

**Keywords:** blood-brain barrier, cerebral blood flow, cerebral vasculature system, neurovascular unit (NVU), neurovascular coupling (NC), pathogenesis and therapy

The neurovascular unit (NVU) is a novel concept which refers to the dynamic multicellular complex and functional interactions between brain tissues and blood vessels (Schaeffer and Iadecola, 2021). Neurons, perivascular astrocytes, microglia, pericytes, and endothelial cells (ECs), as well as the basement membrane (BM) form a functional NVU complex (Bell et al., 2020). These components interact with each other in order to maintain the physiological integrity of the NVU. It is suggested that the NVU modulates neurovascular coupling (NVC) and maintains blood–brain barrier (BBB) functions, which are vital to brain homeostasis and functions (Bell et al., 2020). In NVC, the cerebral blood flow (CBF) and cerebral nutrition supplement demand are closely linked *via* molecular events to regulate the intraluminal diameter of cerebral blood vessels (Bell et al., 2020). The other major function of the NVU is to maintain BBB barrier functions, leading to formation of a specific circumstance for cerebral function and development (Bell et al., 2020). Under physiological conditions, NVU component cells and the BM cooperate in multicellular complex for BBB development and maintenance (Xu et al., 2019; Bell et al., 2020).

Increasing evidence suggests that dysfunction of the NVU can be associated with Alzheimer's disease (AD), Parkinson's disease (PD), sleep disorders, neurovascular diseases, and traumatic brain injury (Schaeffer and Iadecola, 2021). Recent studies demonstrate that amyloid β (Aβ) disrupts cerebral circulation and disturbs capillary blood flow distribution through targeting pericytes (Schaeffer and Iadecola, 2021). The hyperphosphorylated tau, the other main pathogenic factor of AD, selectively inhibits artery dilatation in the process of NVC, contributing to neurodegeneration in AD (Schaeffer and Iadecola, 2021). Studies suggest that dysfunction of NVC and disturbance of cerebral blood flow are also implicated in the pathogenesis of PD, sleep disorders, traumatic brain injury, and other cerebral disorders (Schaeffer and Iadecola, 2021). We still know little about the pathophysiological roles of the NVU in human neurological diseases. In this issue, three articles provide new insights which advance our understanding of the pathophysiological role of the NVU in neurological disorders (Kisler et al.; Rust et al.; Schaeffer and Iadecola, 2021).

The first study by Kisler et al. highlighted that ablation of pericytes can disturb the NVC process in transgenic mice models. Drug-induced loss of pericyte coverage of cortical capillaries was associated with a significant decrease of stimulus-induced CBF responses. Their findings confirm the vital role of pericytes in modulating the NVC process, which suggests that they may play a part in neurological conditions associated with acute or chronic pericyte depletion (such as hypoperfusion and stroke, as well as human neurodegenerative conditions such as AD).

In the second study by Rust et al., the authors developed a fast, automated, and highly reproducible protocol [utilizing the open source software Fiji (ImageJ)] for quantitative analysis and monitoring of various vascular parameters and their alterations. Their novel method provides a practical and reliable guide to monitor dynamic changes in cerebral NVC and the vasculature system under various pathological and physiological conditions in mice and humans, including pharmacological drug-induced alteration of NVC or the cerebral vasculature system.

In the third study, Schaeffer and Iadecola highlighted that dysfunction of the BBB can be associated with neurodegeneration in PD. The increased permeability of the BBB was identified in advanced PD, which was associated with leakage of red blood cells (RBCs) and neurotoxic factors from circulation into parenchymal brain tissue (Schaeffer and Iadecola, 2021). The penetration of RBCs into the brain can cause accumulation of iron species and generation of neurotoxic reactive oxygen species (ROS) (Schaeffer and Iadecola, 2021). In a recent study, Yang et al. studied 204 PD patients and 204 aging healthy controls (HCs), and found that serum SOD level was decreased, while serum hsCRP, an inflammation marker, increased in PD patients. Furthermore, abnormalities in lipid metabolism with decreased cholesterol, HDL-C, and LDL-C were identified in PD, compared with HCs. Plasma HDL and cholesterol were closely related to the integrity of the BBB, and dyslipidemia can cause BBB impairment (Bowman et al., 2012). These findings suggest a link between BBB impairment and PD pathogenesis.

While the findings of the three studies have provided additional pathophysiologic insights, the exact role of the NVU in neurodegenerative diseases still remains to be elucidated. Recent findings demonstrate the molecular heterogeneity of brain NVU cells, suggesting no prototypical neurovascular unit may exist at distinct levels of cerebral NVU networks (Schaeffer and Iadecola, 2021). The cerebral NVU network seems to be more complicated than we expected. Further studies from *in vitro* to *in vivo* models are needed to investigate the role of the NVU in the pathogenesis of various neurological diseases with the hope of identifying potential therapeutic targets.

## Author Contributions

All authors contributed to conceptual design, writing, and approval of the manuscript.

## Funding

This study was supported by the Singapore National Medical Research Council (NMRC; STaR and SPARK II Programe, OF PD LCG 0002).

## Conflict of Interest

The authors declare that the research was conducted in the absence of any commercial or financial relationships that could be construed as a potential conflict of interest.

## Publisher's Note

All claims expressed in this article are solely those of the authors and do not necessarily represent those of their affiliated organizations, or those of the publisher, the editors and the reviewers. Any product that may be evaluated in this article, or claim that may be made by its manufacturer, is not guaranteed or endorsed by the publisher.

## References

[B1] Bell A. H. Miller S. L. Castillo-Melendez M. Malhotra A. (2020). The neurovascular unit: effects of brain insults during the perinatal period. Front Neurosci. 13:1452. 10.3389/fnins.2019.01452 32038147 PMC6987380

[B2] Bowman G. L. Kaye J. A. Quinn J. F. (2012). Dyslipidemia and blood-brain barrier integrity in Alzheimer's disease. Curr Gerontol Geriatr Res. 2012:184042. 10.1155/2012/184042 22654903 PMC3359662

[B3] Schaeffer S. Iadecola C. (2021). Revisiting the neurovascular unit. Nat Neurosci. 24, 1198–1209. 10.1038/s41593-021-00904-7 34354283 PMC9462551

[B4] Xu L. Nirwane A. Yao Y. (2019). Basement membrane and blood-brain barrier. Stroke Vasc. Neurol. 4, 78–82. 10.1136/svn-2018-000198 31338215 PMC6613871

